# Clear Aligner Treatments in Orthoperio Patients

**DOI:** 10.1155/2022/8932770

**Published:** 2022-02-14

**Authors:** Gianluca Mampieri, Roberta Condò, Giovanni Di Caccamo, Paola Pirelli, Aldo Giancotti

**Affiliations:** ^1^Department of Clinical Sciences and Translational Medicine, University of Rome Tor Vergata, Italy; ^2^Private Practice, Rome, Italy

## Abstract

**Introduction:**

Orthodontic treatment is a recognized approach to support specific periodontal issues thanks to its capability to manipulate periodontal tissues. This concept is certainly not new, but the use of aligners in certain clinical conditions can be considered as being innovative when a multidisciplinary treatment is necessary. Moreover, aligners enable to plan 3D tooth movements, root placement, staging, and range of dental movements, alongside the improvement of oral hygiene. Thus, aligners can be suitable for the treatment of periodontal issues. In this article, the authors present two clinical cases with different periodontal issues: one with superficial periodontal problems and the other with a deep one. Both cases were successfully treated with aligners, highlighting how this invisible and comfortable tool can simplify the management of complex adult treatments.

**Conclusion:**

Digital workflow is the key for success in the aligner technique. The possibility to design a virtual plan of treatment and to transfer it in the real clinical world represents a way to limit errors and to reduce the time of orthodontic therapy.

## 1. Introduction

Clear aligner treatment (CAT) is an orthodontic technique to align teeth by means of removable, comfortable, and scarcely visible appliances. Because of these features, the mentioned technique has been recommended since its introduction for the treatment of adult patients. Epidemiological studies have shown that more than 75% of adults have periodontal problems [1], the most common consisting of dental migration, dental extrusion, spacing of incisors, and gingival recessions.

Such patients can be orthodontically treated if the periodontal disease has been kept under control. Nevertheless, when we plan movements of teeth with reduced periodontal support, it is crucial to consider some important factors, such as range of the tooth movements and magnitude of orthodontic forces.

Moreover, we also know that CAT advantages over fixed appliances consist of good aesthetics, ease of use, comfort, and better oral hygiene [2–5]. However, in periodontal patients, another advantage is 3D control and accurate staging of the tooth movements that allow placing the teeth strategically in order to manipulate the periodontal tissues, thus minimizing reaction forces on the adjacent teeth [6].

In addition, it is possible to identify two types of periodontal issues: superficial and deep periodontal problems. The former typically feature an altered gingival smile line and gingival recessions. The latter are characterized by the incidence of infrabony defects.

Management of periodontal issues requires a careful assessment by a team consisting of an orthodontist, a periodontist, and a dental hygienist in order to define the best treatment strategy and proper timing for tooth movement.

Hence, our article evidences how CAT can be an effective and suitable option to treat periodontal issues by means of proper 3D planning of dental movements. ClinCheck® Pro (Align Technology, San Jose, CA, USA), the software to program dental movements of Align Technology, represents the core of the Invisalign technique and the key for a great treatment, but it needs a learning curve to transform virtual planning into a real clinical result.

The innovation in this approach is using a proactive system able to reduce stress for periodontal tissues and the time of treatment.

## 2. Case Report 1

### 2.1. Diagnosis

**Figure 1:**
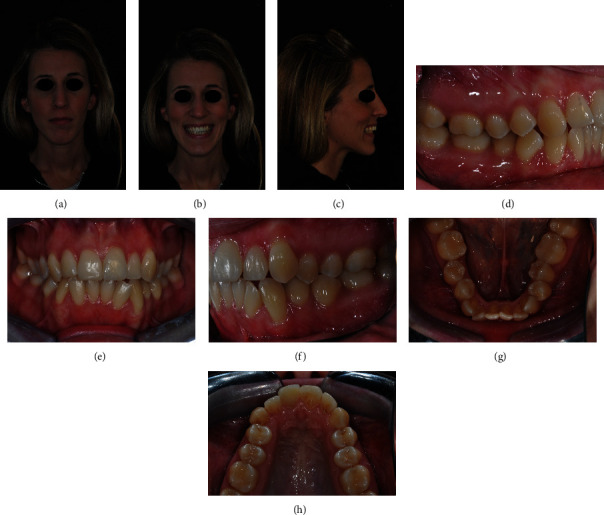
(a–h) Initial clinical records.

**Figure 2:**
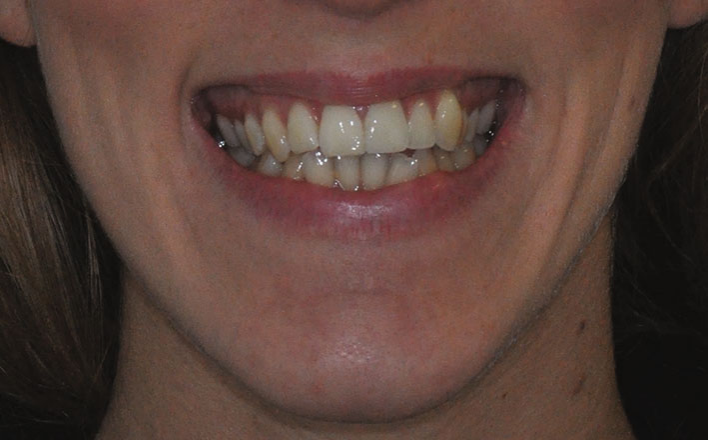
Frontal view with altered gingival smile line; it's possible to note an excessive presence of gingiva in the lateral sectors.

A 21-year-old female came to the orthodontic clinic asking for smile improvement because she complained about the presence of excessive gingiva exposure upon smiling. She presented a Class II subdivision malocclusion, severe crowding in the lower arch, and a narrow upper arch with an evidently altered gingival smile line (Figures 1(a)–1(h)). Indeed, upon frontal view, gingival parabolas of the molars and premolars in the upper arch were more evident than gingival parabolas of the anterior teeth (Figure 2).

The maxillary midline was coincident with the face and with the mandibular midline.

**Figure 3:**
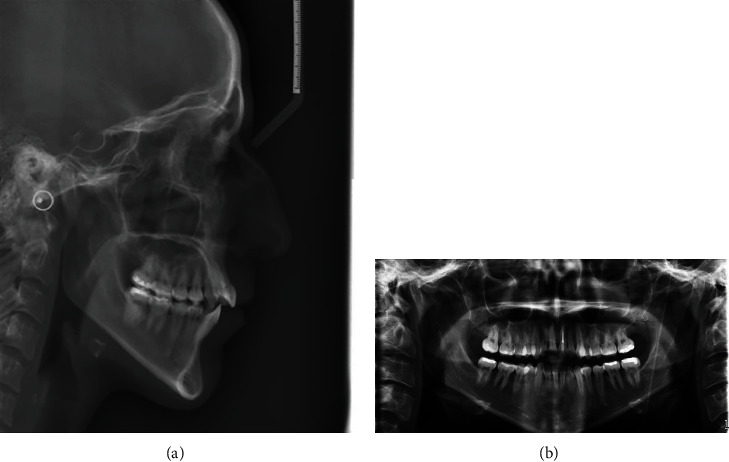
(a, b) Initial radiographic records.

Subsequently, the cephalometric analysis evidenced a skeletal biretrusion, increased mandibular plane angle, increased lower anterior facial height and retrognatic mandible. Panoramic X-ray showed that an endodontic treatment on 2.1 had previously been performed, but no third molars were present (Figures 3(a) and 3(b)).

Furthermore, there were some recordable periodontal alterations that may have affected the orthodontic treatment:

- a thin biotype especially in the lower arch on incisors and cuspids
- gingival recessions on 3.3, 3.2, and 4.4

### 2.2. Treatment Objectives

Treatment objectives were to

1. align and level both arches
2. improve sagittal relationship and occlusion
3. avoid worsening of the periodontal conditions on lower anterior teeth
4. improve aesthetics by reducing the gummy smile and correcting the gingival smile line

### 2.3. Treatment Alternatives

Given that the periodontal conditions revealing lower incisors and cuspids were positioned on the border of the alveolar crest, the alternative treatment would include the use of a fixed appliance, with first bicuspid extraction in both arches and skeletal anchorage by using miniscrews in the lower arch. TADs positioned distally to lower canines would allow for proper extraction space management so as to align anterior teeth without retroclination, while avoiding to increase gingival recessions and to cause distress to the thin bone around the cuspids. Hence, the planned strategy would favor the mesial protraction of posterior teeth after incisor alignment in order to close residual spaces.

However, the aforementioned solution would require high control of the biomechanics as well as experienced clinicians to achieve a valuable result.

From a periodontal point of view, a surgical approach by connective graft on lower incisors and canines would also have been strongly indicated prior to orthodontic treatment, yet due to the thin biotype, this procedure would have also presented some challenges including the high risk of recession worsening. Moreover, the patient was reluctant to fixed and visible appliances.

Thus, the clear aligner option was considered to be the most suitable one for its multiple advantages (comfort, aesthetics, noninvasiveness, nonsurgical approach).

### 2.4. Treatment Plan

The orthodontic treatment with aligners would include 2 phases:

- *Phase I*: slight expansion of the arches; lingual tip correction of lower molars and bicuspids; alignment of lower incisors by IPR; mesial tipping of 3.2-3.1, without moving canines
- *Phase II*: use of additional aligners for distal rotation and extrusion of 3.5; extrusion of 2.5; optimization of aesthetics and occlusion

### 2.5. ClinCheck Virtual Planning

**Figure 4:**
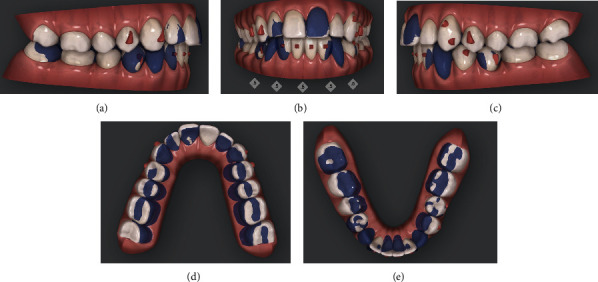
(a–e) Initial/final virtual superimposition of the first phase of aligners: 3.3 and 4.3 blocked to avoid any side effect on the surrounding periodontal tissues.

The first step consisted of the use of 14 aligners in the upper arch and 25 in the lower one (Figures 4(a)–4(e)).

Our objectives were to gain further space in the upper arch, aligning teeth by slight arch expansion, molar derotation, and mild incisor proclination.

In the lower arch, the periodontal issues required careful staging of movements:

1. 3.3 and 4.3 were blocked to avoid any side effect on the surrounding periodontium
2. Intercanine diameter was maintained
3. Incisor alignment was planned by mesial tipping of 3.1 and 3.2 with slight positive torque to improve root position within the alveolar bone
4. IPR was performed from mesial 3.4 to mesial 4.4 to obtain further space
5. Lingual tip correction of the molars and premolars was planned to gain space for alignment
6. Molar intrusion was planned to control vertical dimension

Upon leveling and coordination of both arches, only optimized attachments were used.

The second ClinCheck phase was designed to refine some occlusal aspects, to level both arches, and to correct the left open bite generated in the last stages of the first treatment.

The second phase comprised 17 aligners. In order to correct the left open bite, it was decided to program active forces by using interarch elastics from the bonded aesthetic button on the surface of 2.5 to the metal button on 3.5 to allow extrusion of the former and distal rotation/extrusion of the latter.

**Figure 5:**
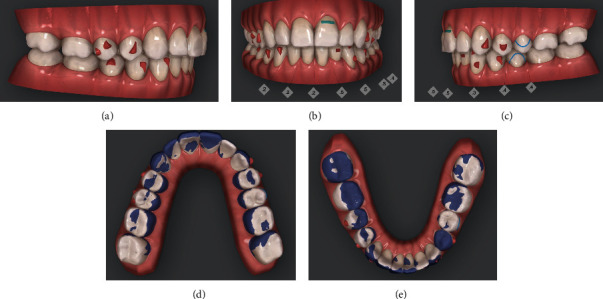
(a–e) Initial/final virtual superimposition of the second phase of aligners.

Further actions involved an additional IPR from mesial 4.4 to mesial 3.6 to gain space and enable slight movements of the lower canines to optimize occlusal contacts (Figures 5(a)–5(e)).

During this last stage, optimized attachments were once again preferred.

All in all, the aligners were 31 in the upper arch and 42 in the lower arch.

### 2.6. Treatment Progress

**Figure 6:**
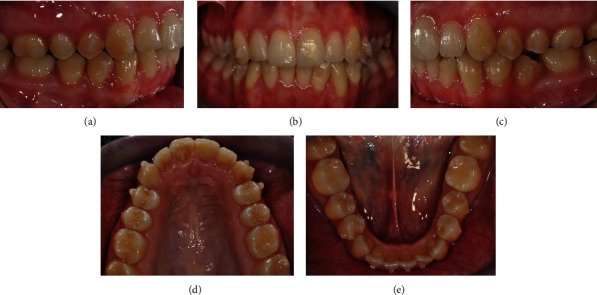
(a–e) Clinical condition at the end of phase I.

As aforementioned, the first phase included 14 aligners in the upper arch and 25 in the lower one. The aligners were changed every 10 days. Upon completion of phase I, we observed that the virtual planning and clinical results were not perfectly superimposable. The clinical situation showed incomplete alignment in both arches, 3.1 and 3.2 maintaining distal tipping, no leveling in either arch, and dental Cl. II on the right side (Figures 6(a)–6(e)).

**Figure 7:**
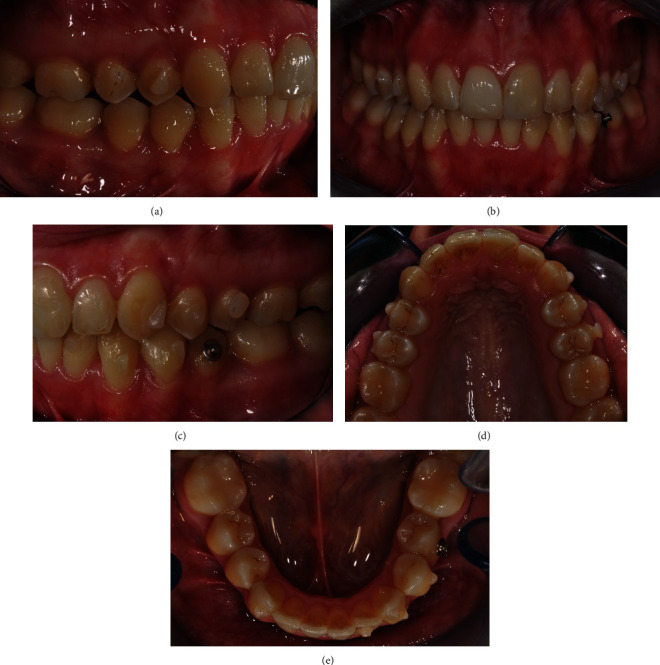
(a–e) Additional aligners with active elastic forces.

The second phase (additional aligners) featured leveling and coordination of both arches, planned to improve the sagittal relationship by means of mesial shift of left mandibular teeth. Moreover, such phase was characterized by the use of active elastic forces to achieve derotation and extrusion of 3.5. The patient was required to wear elastics at least 12 hours/day (Figures 7(a)–7(e)).

### 2.7. Treatment Results

**Figure 8:**
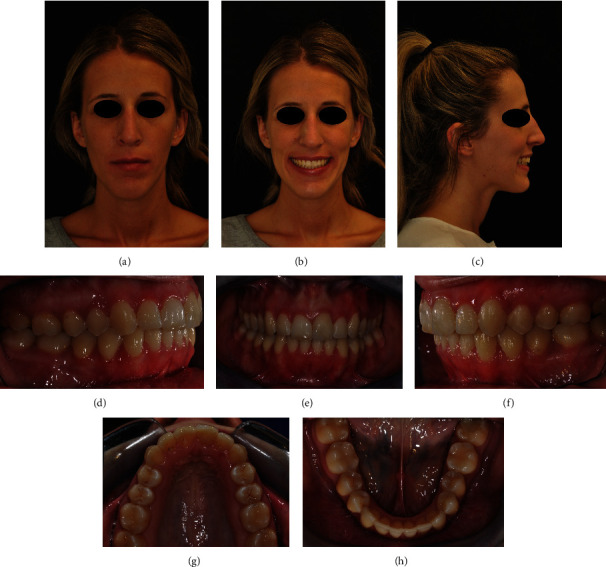
(a–h) End-of-treatment clinical observations.

Final clinical records show a proper bilateral Class I relationship and correct overbite and overjet. A mutually protected occlusion with canine guidance during excursive movements was achieved (Figures 8(a)–8(h)).

Indeed, the patient's smile was esthetically improved. The gummy smile was corrected by leveling the gingival smile line, placing the upper gingival parabolas of the canines at the same height of the central incisors. Frontally viewed, smile aesthetics appeared as being more pleasant, and the result was achieved thanks to torque correction of upper posterior teeth that made gingival parabolas of the molars and premolars less visible than they were initially.

**Figure 9:**
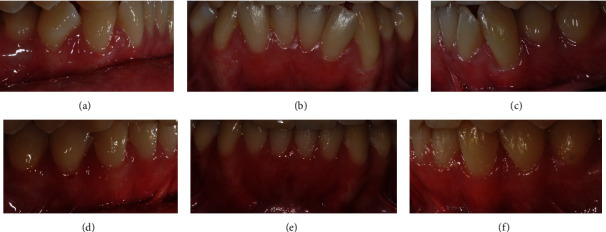
(a–f) Pre-/posttreatment comparison of periodontal tissues.

Moreover, periodontal tissues around lower anterior teeth properly responded. At the end of the treatment, the improved gingival smile line in the lower arch allowed to observe healthy gingival tissues with pale pink and firm gingivae and improvement of gingival recessions probably determined by dental movement favoring tissue manipulation and creeping attachments (Figures 9(a)–9(f)).

**Figure 10:**
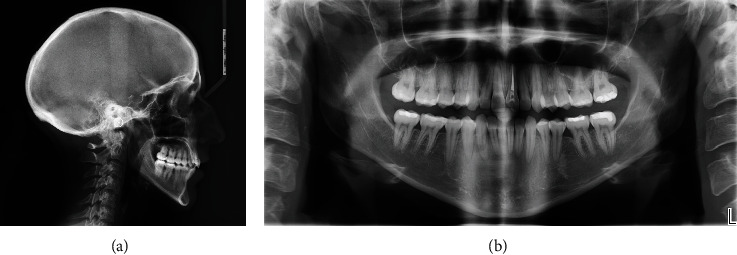
(a, b) Final X-ray records.

As can be seen in the cephalometric superimposition, the sagittal skeletal relations were maintained whereas the vertical skeletal relations slightly changed by 2°. From a vertical point of view, maxillary inclination remained unchanged, while mandibular inclination slightly changed by +2°. Concerning dentobasal relations, the maxillary incisor inclination dropped by 5° by uprighting the upper incisors. Moreover, mandibular incisor inclination was reduced by 2°, thus contributing to mandibular incisor compensation. In terms of dental relations, values show an overjet reduction by 3 mm and a positive change in overbite (from 1 to 3 mm). The interincisal angle also improved by 15°, shifting from 125° to 140° (Figures 10(a) and 10(b); Table 1).

**Figure 11:**
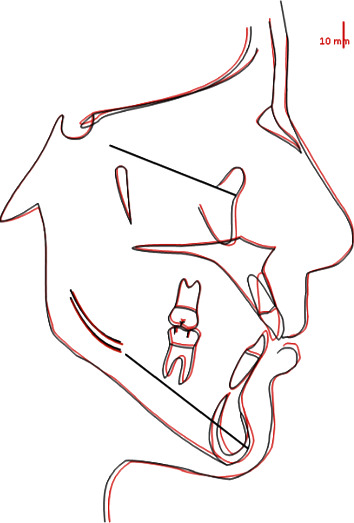
Pre-/postsuperimposition.

Upper molar position maintenance is identified by clinicians as the “bite-block effect” and favors not only the correction of anterior open bite but also an improvement of the Class II relationship (Figure 11; Table 1).

At the end of the treatment, customized thermoformed retainers were provided to the patient in order to promote long-term stability of results [7].

## 3. Case Report 2

### 3.1. Diagnosis

**Figure 12:**
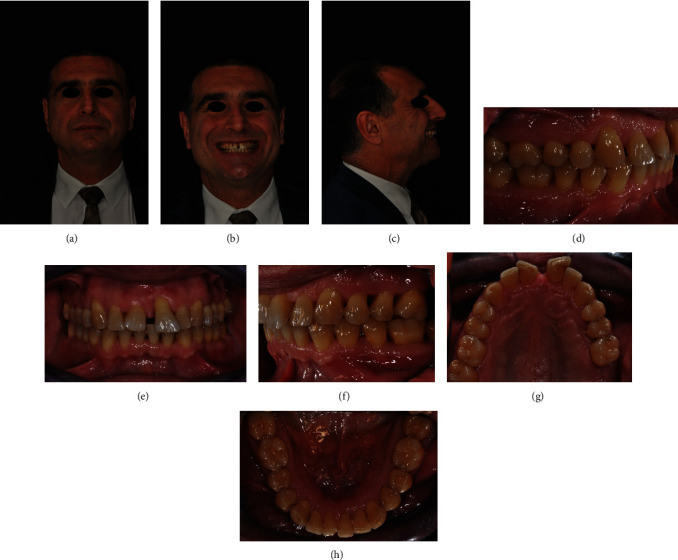
(a–h) Initial clinical records.

A 56-year-old male patient presented an unpleasant smile, featuring a large diastema between upper incisors and a severe proclination of 2.1. Clinical extraoral examination showed a retrognatic mandible. Intraoral examination evidenced Class I relationship on both sides, an excessive incisor proclination, distal tipping and extrusion of 2.1, and gingival recession thereof (Figures 12(a)–12(h)). The patient had previously been treated for chronic periodontal disease by scaling and root planning, and for this reason, 2.7 had been extracted. Upon initial assessment, periodontal probing and periapical X-ray showed an 11 mm infrabony pocket on the mesial side of 2.1 associated with grade 2 dental mobility.

**Figure 13:**
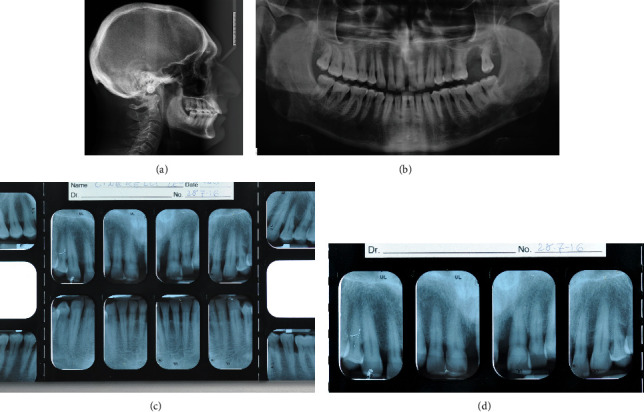
(a–d) Initial radiographic records.

Lateral X-ray analysis showed a mandibular retrusion with a regular mandibular plane angle. Panoramic X-ray evidenced the presence of all third molars. A full-mouth periapical X-ray presented clear aspects of periodontal chronic disease with generalized lack of bone (Figures 13(a)–13(d)).

### 3.2. Treatment Objectives

Treatment objectives were to

1. inactivate the periodontal disease by initial causal therapy with scaling and root planning
2. maintain the sagittal relationship
3. favor mesial tipping of 1.1 and 2.1 to solve the interincisor diastema
4. perform intrusion and retrusion of central incisors by optimizing their position in the alveolar bone
5. perform surgery to improve recession on 2.1 following the orthodontic treatment

### 3.3. Treatment Alternatives

Given the regular occlusal relationship and the issues limited to anterior aesthetics and functionality, no alternative plans were considered for the patient.

An alternative orthodontic technique to aligners would have been traditional fixed appliances.

However, we selected aligners for a number of reasons:

- The possibility to plan individual movements for each tooth
- The need to maintain the occlusal posterior relationship
- The possibility to avoid further periodontal stress by selecting a minimal movement range (0.2 mm/1° per aligner)

### 3.4. Treatment Plan

The orthodontic treatment with aligners would include only one phase:

- Slight coordination of both arches; mesial tipping of central incisors; with intrusion and retrusion thereof; retrusion of lower incisors with IPR

### 3.5. ClinCheck Virtual Planning

**Figure 14:**
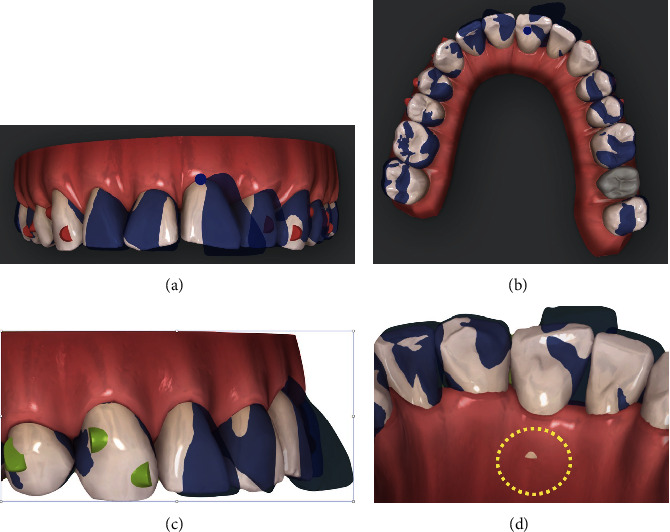
(a–d) Initial/final virtual superimposition with the visualization of slight transparency of the 2.1 root, for torque overcorrection.

According to the ClinCheck® virtual plan, the treatment would consist of 27 aligners (Figures 14(a)–14(d)).

Regarding the upper arch, the objectives would be to correct distal tipping of central incisors, to reduce incisor proclination, and to close the diastema.

The infrabony pocket on the mesial side of 2.1 would require careful staging of movements in order to

1. reduce the range of movement of 2.1, minimizing periodontal tissue stress
2. correct distal tipping meanwhile performing intrusion/retrusion of central incisors
3. plan overcorrection of the palatal torque of 2.1 by placing the root beyond the center of the alveolar crest, 1° of torque for every aligner. For realizing this condition, it is strategic to see on the palatal view of the ClinCheck a slight transparency of the 2.1 root. This prescription should favor natural improvement of gingival recession by means of creeping attachment

Leveling and coordination of both arches were also planned. Only optimized attachments were selected for this treatment.

### 3.6. Treatment Progress

The orthodontic treatment started 3 months after nonsurgical periodontal treatment as established by periodontal guidelines [8–10].

The treatment consisted of 27 aligners. The aligners were changed every 10 days. During treatment, occlusal relationships were kept optimal, and taking advantage of the posterior anchorage, the intrusion of incisors was programmed without any complications.

During orthodontic treatment, the patient received regular supportive periodontal care (maintenance) undergoing professional hygiene every 6 weeks. Such maintenance protocol was meant to maintain high levels of oral hygiene avoiding side effects of tooth intrusion with infrabony defects. Indeed, tilting and intrusion of plaque to a subgingival position could induce an apical shift of the connective tissue attachment and development of infrabony pockets [11, 12].

### 3.7. Treatment Results

**Figure 15:**
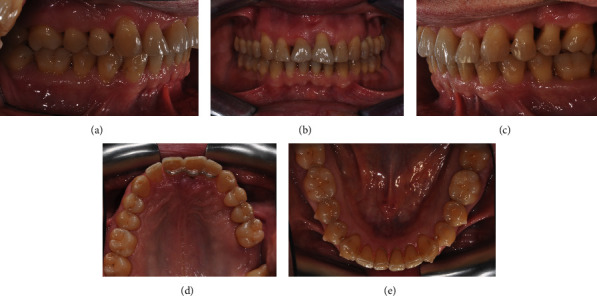
(a–e) Final clinical records.

At the end of the orthodontic treatment, virtual planning and clinical results were quite superimposable. Clinical conditions showed that the Class I canine and molar relationship was preserved, and complete intrusion and alignment of upper incisors with healthy gingival tissues was reached. Upper incisors were splinted by a customized bonded wire in order to limit the use of thermoformed retainers only to nighttime [7] (Figures 15(a)–15(e)).

**Figure 16:**
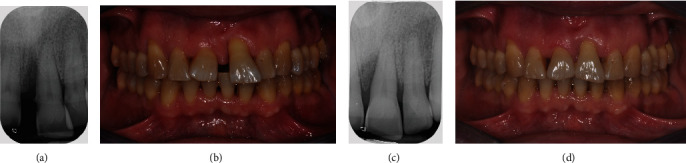
(a–d) Clinical and radiographic differences pre- and immediately postorthodontic treatment.

Esthetically, the patient's smile was so significantly improved that the patient refused to undergo plastic gingival surgery and veneering on the upper incisors (Figures 16(a)–16(d)).

**Figure 17:**
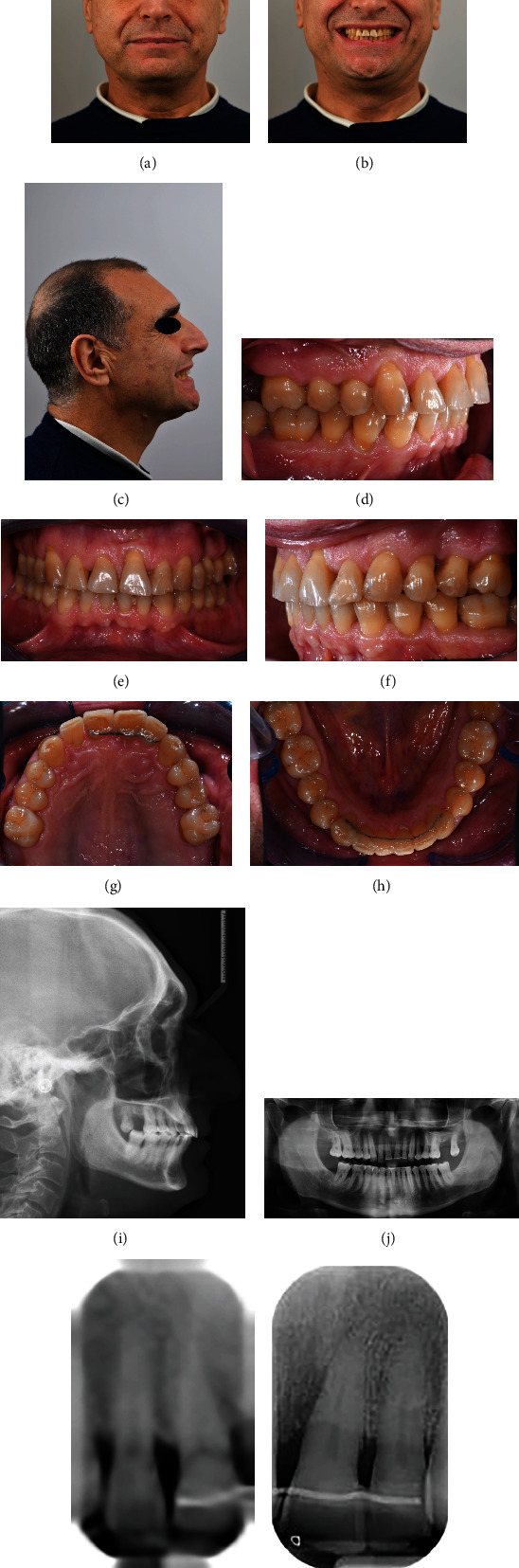
(a–m) Follow-up after 24 months.

Clinical examination at 24-month follow-up and posttreatment full-mouth periapical radiograph series showed a gradual improvement of superficial and deep periodontal tissues around 2.1. Moreover, it was possible to observe a coronal migration of the gingival tissue around 2.1, probably determined by the creeping attachment phenomenon favored by proper root placement and oral hygiene maintenance (Figures 17(a)–17(n)).

The full-mouth periapical radiographs evidenced an adequate presence of cortical bone profile around 2.1 as well as a buccal and palatal profile of the cortical bone, while clinical probing was physiological and without bleeding.

**Figure 18:**
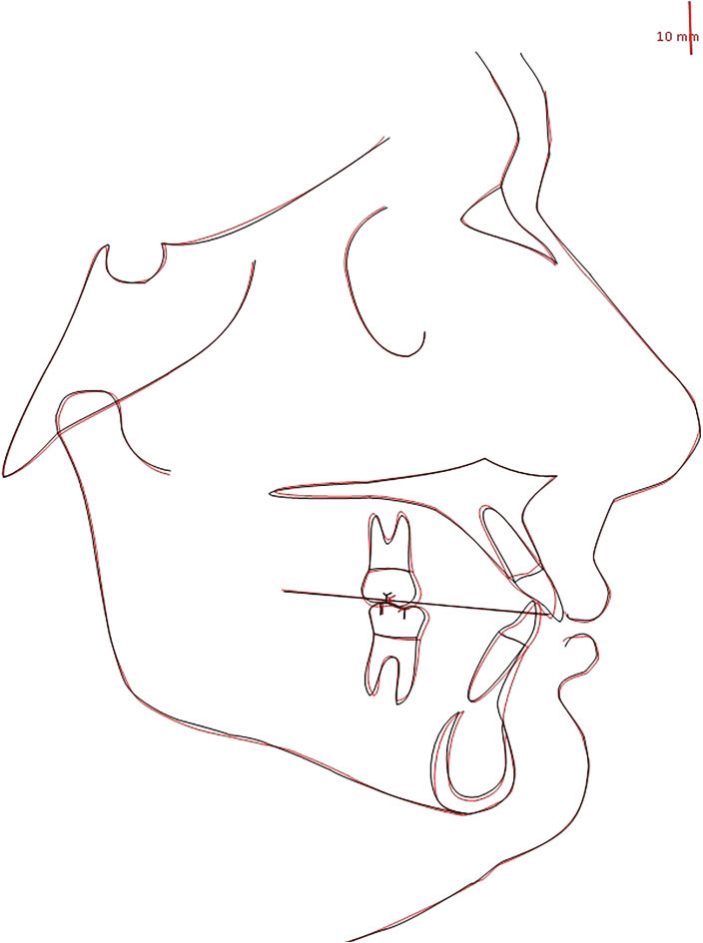
Pre-/postsuperimposition.

As can be seen in the cephalometric superimposition, the sagittal skeletal relations were maintained (Figure 18).

From a vertical point of view, S-N/GO-GN slightly changed by -1°. Hence, maxillary inclination was maintained with a slight change in the mandibular inclination. Moreover, in terms of dentobasal relations, upper incisors were retracted and intruded, thus reducing their inclination by 5°. In addition, mandibular incisor inclination was reduced by 3°. Regarding dental relations, values show an overjet reduction by 2 mm and a positive change in overbite (from 2 to 1 mm). The interincisal angle changed by +8°, thanks to uprighting of upper incisors, shifting from 112° to 120° (Table 2).

## 4. Discussion

In recent years, orthodontists have been paying increasing attention to periodontal issues in terms of orthodontic treatment planning. For this reason, CBCT (Cone Beam Computed Tomography) is a growingly requested X-ray test for a number of reasons:

1. To properly identify the cortical bone features
2. To evaluate the possibility of dental expansion
3. To assess the extent of applicable tip and torque changes

However, due to its ionizing radiation, higher costs, and limited access, CBCT cannot be used as a routine diagnostic method for all patients [13, 14].

Most of the time, clinical examination assisted by conventional X-ray testing is enough to diagnose periodontal issues.

Orthodontic treatment has been considered as a valuable procedure in order to support periodontal treatment aiming at the resolution of specific periodontal issues. Indeed, orthodontics can be a proper means to improve periodontal tissues also by using minimally invasive techniques as aligners.

First of all, innovative diagnostic software can allow staging of minimal dental movements in order to apply light forces, minimizing stress on the periodontal support [14–18].

Secondly, accurate 3D dental movement planning for every single tooth with reference to root placement is favored.

Moreover, the proper use of digital means lowers the risk of worsened periodontal support, particularly in the case of gingival recessions where the uncontrolled buccal movement of the roots could cause damage. Therefore, it is necessary to design dental movements with accurate root control to avoid roots be pushed outside the border of the alveolar crest.

Analyzing clinical Case 1, the digital plan was strategic. As a matter of fact, lower canines were initially blocked during the alignment of incisors to avoid buccal movements of the cuspids, thus avoiding worsening of gingival recessions. Afterwards, during the second series of aligners, the minimal movement of lower canines was planned, with extremely low reactive forces on adjacent teeth. Additionally, adequate staging of dental movements, accurate tip correction, extrusion of 3.2, and increase of lingual torque of 3.3 and 4.3 have redesigned the gingival smile line allowing for an improvement of recessions and better aesthetics [19, 20].

In addition to orthodontic movements, the bacterial plaque should be reduced as much as possible in order to avoid marginal gingivitis, thus favoring gingival tissue creeping attachment.

In the second clinical case, because of reduced periodontal support, digital planning included limited movement of element 2.1 to produce calibrated and well-balanced orthodontic forces. The prescription required tooth movement per aligner equivalent to approximately half the standard one. Moreover, aligner treatment allowed limiting the mobility discomfort initially present on element 2.1. Slow displacement of 2.1 with a rigid control of oral hygiene allowed designing both superficial and deep periodontal frameworks. Periodontal tissues followed tooth movements, remodeling superficial and deep periodontal frameworks as evidenced by clinical and radiographic follow-up (Figures 17(a)–17(i)). Upon consultation, it was decided to avoid a surgical procedure given the good response to the treatment on the whole.

Thus, one can affirm that the intrusion movement in healthy sites does not cause detrimental effects, but if carefully planned and with regular supportive periodontal care (maintenance), it can lead from a long junctional epithelium without any changes to the periodontal ligament attachment level to a new cementum development and collagen attachment [21].

For the described periodontal issues, an aligner-based approach can be valuable as no other orthodontic techniques currently allow to specifically plan the range of movement of every single tooth or to limit periodontal support stress.

## 5. Conclusion

In conclusion, orthodontics represents a proper way to positively modify periodontal tissues. Although the concept was previously well known, our study highlights how aligner treatment can be considered as a valid orthodontic technique to achieve effective results.

The aligner technique comprises features as effective 3D control of tooth movement and root placement, as well as the possibility to plan the staging and range of dental movements, better oral hygiene standards, and safe tooth movements even in cases with reduced periodontal support.

Hence, as it is true that diagnosis and biomechanical planning serve as a basis for orthodontic treatment plan, digital and material development further provides the orthodontist with effective tools that ease management of complex cases.

## Figures and Tables

**Table 1:** Summary of cephalometric values of Case 1.

Cephalometric morphological assessment	Mean SD	Pre	Post
*Sagittal skeletal relations*			
Maxillary position S-N-A	82° + 3.5°	80°	81°
Mandibular position S-N-PG	80° + 3.5°	79°	80°
Sagittal jaw relation A-N-PG	2° + 2.5°	1°	1°
*Vertical skeletal relations*			
Maxillary inclination S-N/ANS-PNS	8° + 3.0°	7°	7°
Mandibular inclination S-N/GO-GN	33° + 2.5°	42°	44°
Vertical jaw relation ANS-PNS/GO-GN	25° + 6.0°	35°	37°
*Dentobasal relations*			
Maxillary incisor inclination 1/ANS-PNS	110° + 6.0°	115°	100°
Mandibular incisor inclination 1/GO-GN	94° + 7.0°	86°	84°
Mandibular incisor compensation 1/A-PG (MM)	2 + 2.0 mm	1.5 mm	1.3 mm
*Dental relations*			
Overjet (mm)	3.5 + 2.5 mm	6 mm	3 mm
Overbite (mm)	2 + 2.5 mm	1 mm	3 mm
Interincisal angle 1/1	132° + 6.0°	125°	140°

**Table 2:** Summary of cephalometric values of Case 2.

Cephalometric morphological assessment	Mean SD	Pre	Post
*Sagittal skeletal relations*			
Maxillary position S-N-A	82° + 3.5°	84°	84°
Mandibular position S-N-PG	80° + 3.5°	81°	82°
Sagittal jaw relation A-N-PG	2° + 2.5°	3°	2°
*Vertical skeletal relations*			
Maxillary inclination S-N/ANS-PNS	8° + 3.0°	7°	7°
Mandibular inclination S-N/GO-GN	33° + 2.5°	28°	27°
Vertical jaw relation ANS-PNS/GO-GN	25° + 6.0°	21°	20°
*Dentobasal relations*			
Maxillary incisor inclination 1/ANS-PNS	110° + 6.0°	118°	113°
Mandibular incisor inclination 1/GO-GN	94° + 7.0°	109°	106°
Mandibular incisor compensation 1/A-PG (MM)	2 + 2.0 mm	1 mm	0.5 mm
*Dental relations*			
Overjet (mm)	3.5 + 2.5 mm	6 mm	4 mm
Overbite (mm)	2 + 2.5 mm	2 mm	1.5 mm
Interincisal angle 1/1	132° + 6.0°	112°	120°
